# Stimulation-induced structural changes at the nucleus, endoplasmic reticulum and mitochondria of hippocampal neurons

**DOI:** 10.1186/s13041-018-0387-2

**Published:** 2018-07-27

**Authors:** Jung-Hwa Tao-Cheng

**Affiliations:** NINDS Electron Microscopy Facility, National Institute of Neurological Disorders and Stroke, National Institutes of Health, Bethesda, MD 20892 USA

**Keywords:** Electron microscopy, ER, Calcium regulation, Chromatin, Nucleus

## Abstract

Neurons exhibit stimulation-induced ultrastructural changes such as increase of thickness and curvature of the postsynaptic density, decrease in contact area between subsurface cistern and plasma membrane, and formation of CaMKII clusters and synaptic spinules. These structural characteristics help in identifying the activity state of the neuron and should be taken into consideration when interpreting ultrastructural features of the neurons. Here in organotypic hippocampal slice cultures where experimental conditions can be easily manipulated, two additional features are documented in forebrain neurons as reliable benchmarks for stimulation-induced structural changes: (1) The neuronal nucleus showed conspicuous clustering of dark chromatin, and (2) the endoplasmic reticulum formed stacks with a uniform gap of ~ 13 nm filled with dark materials. Both structural changes progressed with time and were reversible upon returning the slice cultures to control medium. These stimulation-induced structural changes were also verified in dissociated hippocampal neuronal cultures and perfusion-fixed brains. In hippocampal slice cultures, the neuronal chromatin clustering was detectable within 30 s of depolarization with high K^+^ (90 mM) or treatment with NMDA (50 μM). In contrast, the formation of ER cisternal stacks did not become apparent for another 30 s. Importantly, in dissociated neuronal cultures, when the extracellular calcium was chelated by EGTA, treatment with high K^+^ no longer induced these changes. These results indicate that the stimulation-induced chromatin clustering and formation of ER stacks in neurons are calcium-dependent. Additionally, mitochondria in neuronal somas of tissue culture samples consistently became swollen upon stimulation. However, swollen mitochondria were also present in some neurons of control samples, but could be eliminated by blocking basal activity or calcium influx. This calcium-dependent structural change of mitochondria is specific to neurons. These structural changes may bring insights to the neuron’s response to intracellular calcium rise upon stimulation.

**Electronic supplementary material:**

The online version of this article (10.1186/s13041-018-0387-2) contains supplementary material, which is available to authorized users.

## Introduction

Excitable cells like neurons and muscles show morphological changes under excitatory conditions. Prominent examples include electron microscopy (EM) studies that shed lights on synaptic vesicle recycling in frog neuromuscular junctions [1], and mouse hippocampal neurons in cell cultures [2]. Other EM studies on mouse and rat neurons have shown stimulation-induced increases in the thickness and curvature of the postsynaptic density (PSD) [3, 4], decrease in number and contact areas of subsurface cisterns with the plasma membrane [5], and formation of calcium calmodulin-dependent kinase II (CaMKII) clusters [4, 6] and synaptic spinules [7]. While some of the features require precise quantification to document, the presence of CaMKII clusters and synaptic spinules, in themselves, provides strong evidence that the samples are under stimulated conditions. Although the stimulation protocols employed in these studies are beyond normal physiological conditions and thus may render neurons under excitatory stress, all of these stimulation-induced structural changes are nevertheless reversible. These structural benchmarks are useful in interpreting whether structural characteristics are caused by heightened neuronal activity or other factors such as genetic manipulations. The present study set out to document additional stimulation-induced structural changes that may serve as helpful benchmarks to detect acute excitation in neurons, and to determine whether these effects are calcium-dependent.

One striking feature is manifested as clustering of dark chromatin in neuronal nucleus from rat hippocampal slice cultures within 30 s upon stimulation. Whether this feature is consistently induced by stimulation or hypoxic excitatory stress is examined in rat dissociated hippocampal cultures and in perfusion-fixed rat and mouse brains.

A second stimulation-induced benchmark structure is the stacking of endoplasmic reticulum (ER) with a uniform gap of ~ 13 nm between stacks. In Purkinje neurons of the cerebellum, formation of ER cisternal stacks is induced by hypoxic and hypoglycemic conditions that were caused by a few minutes of delay in perfusion fixation [4, 8]. Here, hippocampal neurons from all three experimental systems were examined to verify whether similar ER stacks form in forebrain neurons upon stimulation. Furthermore, the time course and reversibility of the formation of ER stacks were examined in hippocampal slice cultures.

A third structural change is that mitochondria in neuronal soma became swollen upon stimulation. Similar ultrastructural changes in mitochondria has been reported in rat dissociated hippocampal cultures upon excitotoxic injury [9] and in mice after hypoxic-ischemic brain injury [10]. The present study tested whether the swollen mitochondria are induced by activity and calcium influx.

## Methods

### Preparation, treatment and fixation of rat dissociated hippocampal neuronal cultures

Most samples were from a previously published report [5] and reexamined here for additional structural changes. Briefly, cell cultures were prepared from embryonic 20-day-old rat fetuses by papain dissociation, and then plated on glial feeder cultures, and experiments were carried out with three-week-old cultures. Culture dishes were placed on a floating platform in a water bath maintained at 37 °C. Control incubation medium was HEPES-based Kreb’s Ringer at pH 7.4. High K^+^ medium was at 90 mM KCl. N-methyl-D-aspartic acid (NMDA) medium contained 30–250 μM NMDA in the control medium. APV (50 μM), an NMDA antagonist, was included in the NMDA medium (50 μM) for some experiments. Cell cultures were washed with control medium and treated for 2–3 min with either control or high K^+^ media, or with 2–15 min with NMDA, or 2 min of NMDA+APV. In order to test whether extracellular calcium is involved in stimulation-induced structural changes, a calcium chelator, EGTA (1 mM), was included in a calcium-free control or high K^+^ medium where osmolarity was compensated with sucrose. In some experiments, basal activity of cultures was blocked by 1 h incubation in culture media containing tetrodotoxin (TTX at 0.5 μM) to block action potential, D(−)-amino-7-phosphonovaleric acid (APV at 50 μM) to block NMDA receptor activation, and 6-cyano-7-nitoquinoxaline-2,3-dione (CNQX at 20 μM) to block AMPA receptor activation. Treated samples were then fixed immediately with 4% glutaraldehyde in 0.1 N cacodylate buffer at pH 7.4 for 30 min at room temperature and then stored in fixative at 4 °C.

### Preparation, treatment and fixation of rat organotypic hippocampal slice cultures

Most samples were from a previously published report [7] and reexamined here for additional structural changes. Briefly, the hippocampus was removed from postnatal 6–8-day old rats and cut at 250 μm thickness with a tissue chopper. Slices were placed on a cell culture inserts in six-well culture dishes and used 10-14 days in vitro with the dishes on a floating platform in a water bath at 37 °C. Normal, high K^+^ and NMDA medium were the same as used for dissociated cells. Both high K^+^ and NMDA treatments were for 0.5, 1, 2, 3, or 5 min. To examine recovery after depolarization, high K^+^ medium was removed and the samples were washed three to four times in normal incubation medium for a total of 1, 2, 5, 10, 30 and 60 min. Experimental controls were processed in parallel, including all the medium changes and washing steps. Slice cultures were fixed with 2% glutaraldehyde and 2% paraformaldehyde, or 4% glutaraldehyde in 0.1 N cacodylate buffer at pH 7.4 for 1–3 h at room temperature and then stored at 4 °C.

### Perfusion fixation of rat and mouse brains

Most samples were from previously published reports [4, 6] and reexamined here for additional structural changes. Briefly, adult rats were deeply anesthetized with Nembutal, and mice from 17-day to 3-month-old were deeply anesthetized with isoflurane. Animals were perfusion fixed through the heart with 2% glutaraldehyde + 2% paraformaldehyde in 0.1 M sodium cacodylate buffer at pH 7.4, or first perfused with 3.75% acrolein+ 2% paraformaldehyde, then followed by 2% paraformaldehyde. The time interval starting from the moment the diaphragm was cut to the moment when the outflow from the atrium turned from blood to clear fixative was recorded. Those animals that were successfully perfused within 100 s were classified as “fast” perfusion. For the “delayed” perfusion experiments, phosphate buffered saline containing calcium and magnesium was first perfused through the heart for 5 min before the start of the fixative. Neurons were under basal state after fast perfusion, and under ischemic excitatory conditions after delayed perfusion fixation [4, 6]. The perfusion-fixed brains were dissected and vibratomed into 100 μm thick coronal slices and stored in 2% glutaraldehyde in buffer at 4 °C.

### Electron microscopy

Most fixed samples were washed in buffer and treated with 1% osmium tetroxide in 0.1 N cacodylate buffer at pH 7.4 for 1 h on ice. Some dissociated cells samples were treated with “reduced osmium” (1% osmium tetroxide + 1% potassium ferrocyanide in 0.1 N cacodylate buffer at pH 7.4 for 1 h on ice). Samples were then washed and en bloc stained with 0.25–1% uranyl acetate in 0.1 N acetate buffer at pH 5.0 overnight at 4 °C, dehydrated with a series of graded ethanol, and finally embedded in epoxy resins. Thin sections (70–90 nm thick) were counterstained with uranyl acetate and lead citrate, examined under a JEOL1200EX transmission electron microscope, and photographed with a bottom-mounted digital CCD camera (AMT XR-100, Danvers, MA, USA) at 4–10,000× for nucleus and at 10–40,000× for ER cisternal stacks and mitochondria.

### Morphometry

#### Sampling of neuronal vs. astroglial nucleus

In perfusion-fixed brains and in hippocampal slice cultures, sampling of neuronal nuclei was restricted to the pyramidal cells in the CA1 region of the hippocampus. In dissociated cultures, neurons were mixed with astrocytes; the nuclei of the two cell types can be identified by their distinct ultrastructure [11]. For all three experimental systems, every neuronal soma encountered was photographed at 4–10,000× to document chromatin clustering.

The predominant type of astrocytes in slice cultures is “fibrous” (see [11] for classification of types of astrocytes), and every fibrous astroglial nucleus encountered was photographed until at least 10 astroglial nuclei were imaged per slice culture. More than 132 astroglial nuclei were imaged from 11 samples.

#### Scoring of ER cisternal stacks in neuronal somas

**Fig. 1:**
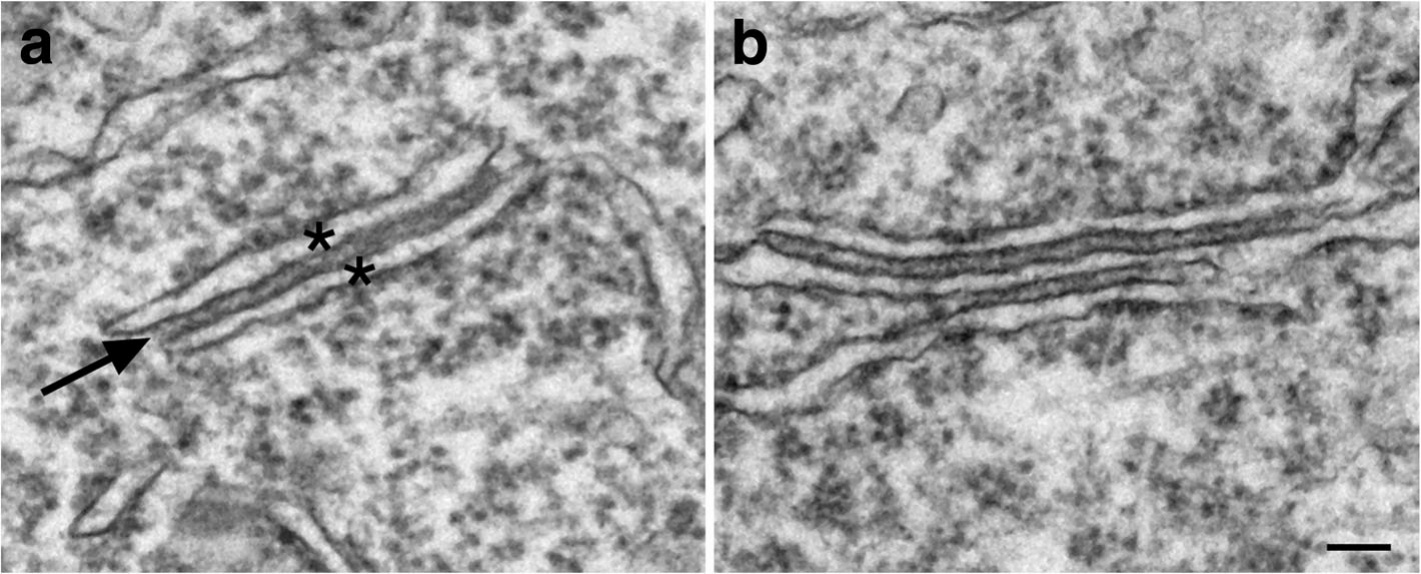
ER cisternal stacks, defined by at least two ER cisterns (asterisks in a) apposed with a uniform ~ 13 nm gap (arrow in a). Images taken from hippocampal slice cultures treated with 3 min of 90 mM high K^+^ (**a**) or 5 min of 50 μM NMDA (**b**). Scale bar = 0.1 μm

In both slice and dissociated cultures, every soma encountered was scored for presence of ER stacks. An ER cisternal stack is defined as two ER cisterns (marked by asterisks in Fig. 1a) closely apposed with a uniform gap of ~ 13 nm (arrows in Fig. 1a), filled with dense material, and the membranes facing the gap are free of ribosomes. Multiple cisterns closely apposed were sometimes present upon more intense stimuli (Fig. 1b). Every ER cisternal stack encountered, whether it consisted of 2 or more cisterns was counted as one entity in the present study. The frequency of ER stacks for each sample was expressed as total number of ER stacks per 100 neuronal somas.

At least 10 somal profiles were scored for each sample, with ~ 600 somas scored from 30 samples of slice cultures and more then 500 somas scored form 25 samples of dissociated cultures. Neurons that were partially under the grid bar were still included in the sampling as long as at least half of the nucleus was visible. Due to the fact that many neurons were scored with partial profiles, the total number of ER stacks from each sample was pooled from all somas that were scored, and then divided by the number of neurons and normalized as number of SSCs per 100 neuronal somas. This practice ensures that each sample is treated the same, resulting in one data point per sample.

#### Scoring of mitochondria morphology in neuronal somas

**Fig. 2:**
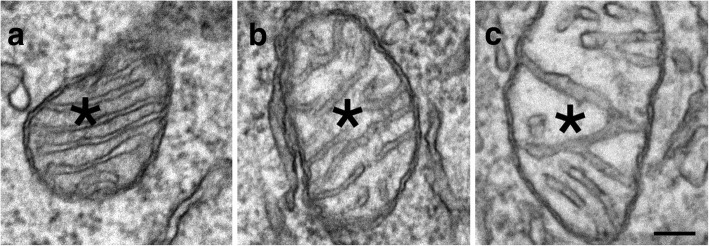
Neuronal mitochondria are classified as “not swollen” if the matrix (marked by *) is dark (**a**), or “swollen” if the matrix is light (* in **b**, **c**). Images taken from dissociated hippocampal cultures treated for 2 min with calcium-free medium containing EGTA (1 mM; **a**), control medium (**b**), and high K^+^ medium (90 mM; **c**). Scale bar = 0.1 μm

Mitochondria morphology was scored in every neuronal soma encountered. Based on the appearance of the matrix (asterisks in Fig. 2a-c), mitochondria were classified as “not swollen” (Fig. 2a) with a dark matrix, or “swollen” with a light matrix, including slightly swollen (Fig. 2b) and extensively swollen (Fig. 2c) ones. A neuron is classified as containing swollen mitochondria if any of its mitochondria appeared swollen. A percentage of neurons containing swollen mitochondria was calculated for each sample.

#### Statistical analysis

Comparisons between two groups were tested by Student’s t test or paired t test. Comparisons among three groups or more were tested by one-way ANOVA with Tukey’s post-test.

## Results

### Stimulation-induced changes in chromatin configuration in neuronal nucleus

**Fig. 3:**
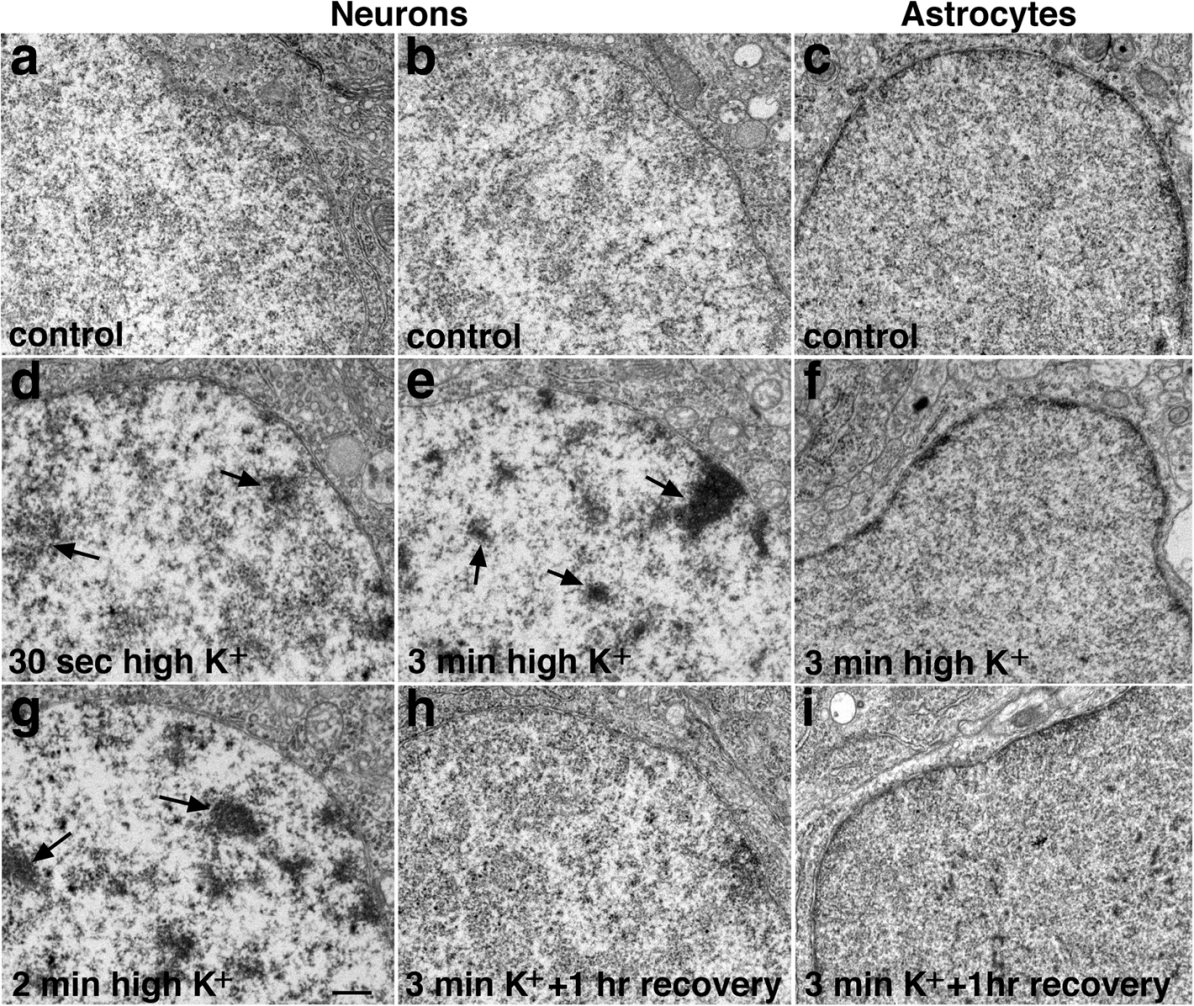
Chromatin in neurons aggregates into dark clusters upon depolarization. Electron micrographs of neuronal nuclei from CA1 region of the hippocampus in slice cultures under different experimental conditions. (left column) – Chromatin appeared non-clustered under control conditions (**a**), but became aggregated into dark clusters (arrows) at 30 s after depolarization with high K^+^ (90 mM, **d**), and progressed into even more pronounced clustering after 2 min of treatment (**g**). (middle column) – In a second series of experiments, the chromatin was again non-clustered under control conditions (**b**), became extensively clustered upon 3 min of depolarization with high K^+^ (**e**), and reverted to the non-clustered appearance upon washout of the high K^+^ medium and returning the samples to control medium for 1 h (**h**). (right column) – In contrast, nucleoplasm of fibrous astrocytes had a thin but dense rim of dark material of irregular thickness around the edge of the nucleus, and this configuration was not affected by high K^+^ (**c**, **f**, **i**). Scale bar = 0.5 μm

One striking feature of acutely stimulated neurons was the appearance of clusters of dark chromatin in the nuclei. Figure 3 shows the nuclei of neurons and astrocytes in the CA1 region of the hippocampus in organotypic slice cultures. Under control conditions, the nucleoplasm of the pyramidal neurons had a non-clustered appearance at low magnification (Fig. 3a) in all 15 samples examined. Upon depolarization with high K^+^ (90 mM), chromatin became clustered as dark aggregates (arrows in Fig. 3) in all 13 samples examined. This clustering of chromatin progressed with treatment time, from a less dense appearance at 30 s (Fig. 3d) to a more condensed form at 2 min (Fig. 3g) of treatment, and with lighter areas interspersed among the dark chromatin clusters. NMDA treatment (50 μM) induced a similar clustering of dark chromatin, which also progressed with treatment time in all 14 samples examined (Additional file 1).

This chromatin reorganization was reversible, as the appearance of the clustered chromatin upon depolarization (Fig. 3e) reverted back to that similar to the control conditions (Fig. 3b) upon cessation of stimulation and recovery in control medium (Fig. 3h). At least 10 min recovery in control medium was needed for the nucleus to revert back to its non-clustered appearance after a 3 min high K^+^ treatment. In another experiment, at 5 min recovery after a 1 min high K+ treatment, the chromatin was still somewhat clustered. This stimulation-induced clustering of chromatin is specific to neurons, as astroglial nuclei (Fig. 3c, f, j) from the same slice cultures did not show similar chromatin clustering upon depolarization (consistent in 4 experiments; Additional file 2).

**Fig. 4:**
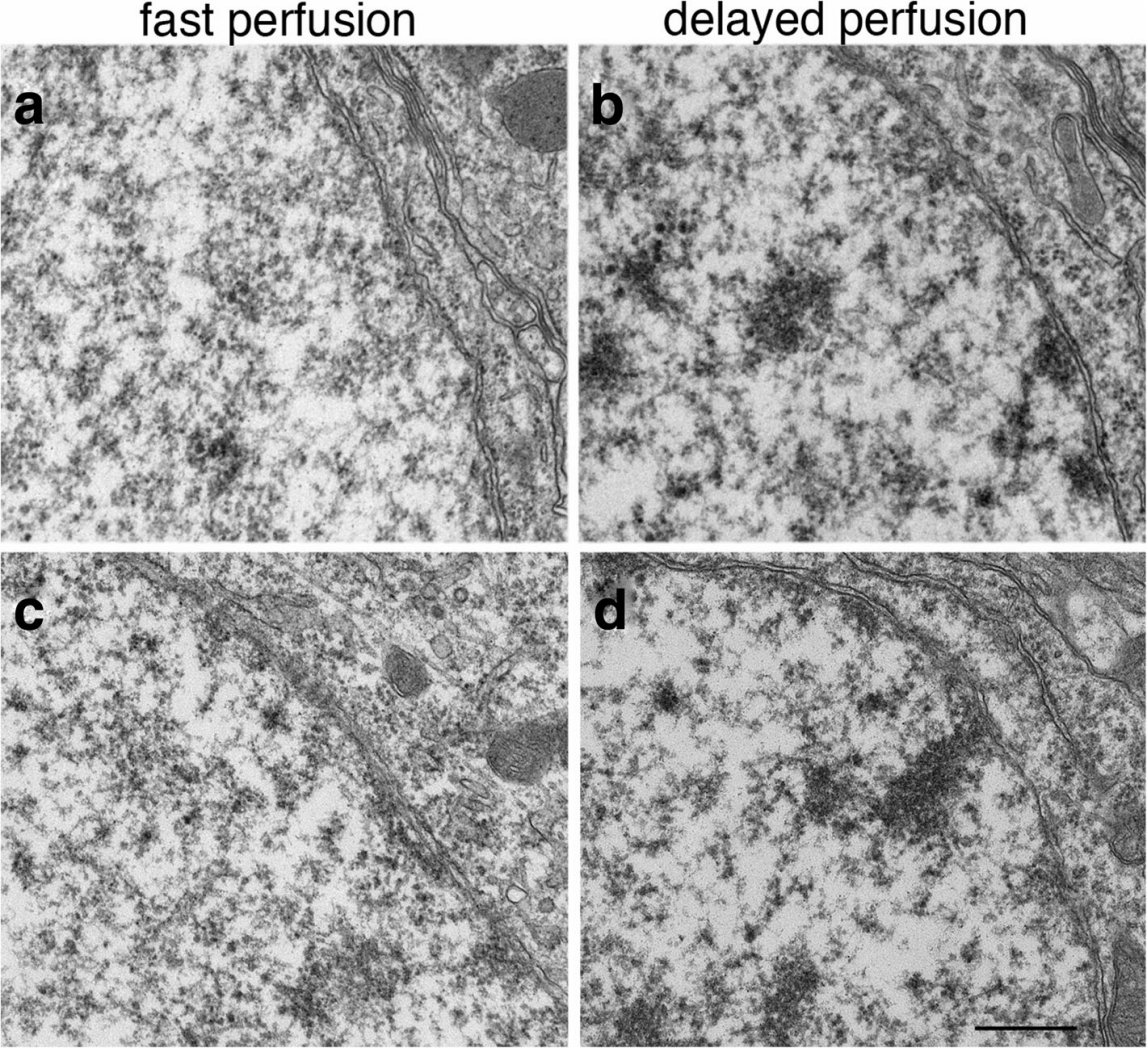
Chromatin appeared non-clustered in fast perfusion-fixed mouse brains where neurons are under a basal state (**a**, **c**), but aggregated into dark clusters in delayed perfusion-fixed brains where neurons are under excitatory stress (b, d). Samples were from the CA1 region of the hippocampus (**a**, **b**) and layer III of cerebral cortex (**c**, **d**). Scale bar = 0.5 μm

This stimulation-induced chromatin clustering was also observed in other experimental systems. In fast perfusion-fixed mouse or rat brains, where neurons are presumed to be under basal conditions [4], the nuclei of neurons in 13 animals had a non-clustered appearance (Fig. 4a, c), consistent with images shown in a classic neurocytology atlas [11] where brains were optimally perfusion-fixed. In contrast, in 7 delayed perfusion-fixed brains, where neurons were under hypoxic excitatory stress [4], chromatin became aggregated as dark clusters (Fig. 4b, d) in different regions of the brain, including pyramidal neurons in the CA1 region and granules cells of the dentate gyrus of the hippocampus, pyramidal neurons in layer III of the cerebral cortex, and Purkinje cells of the cerebellum.

**Fig. 5:**
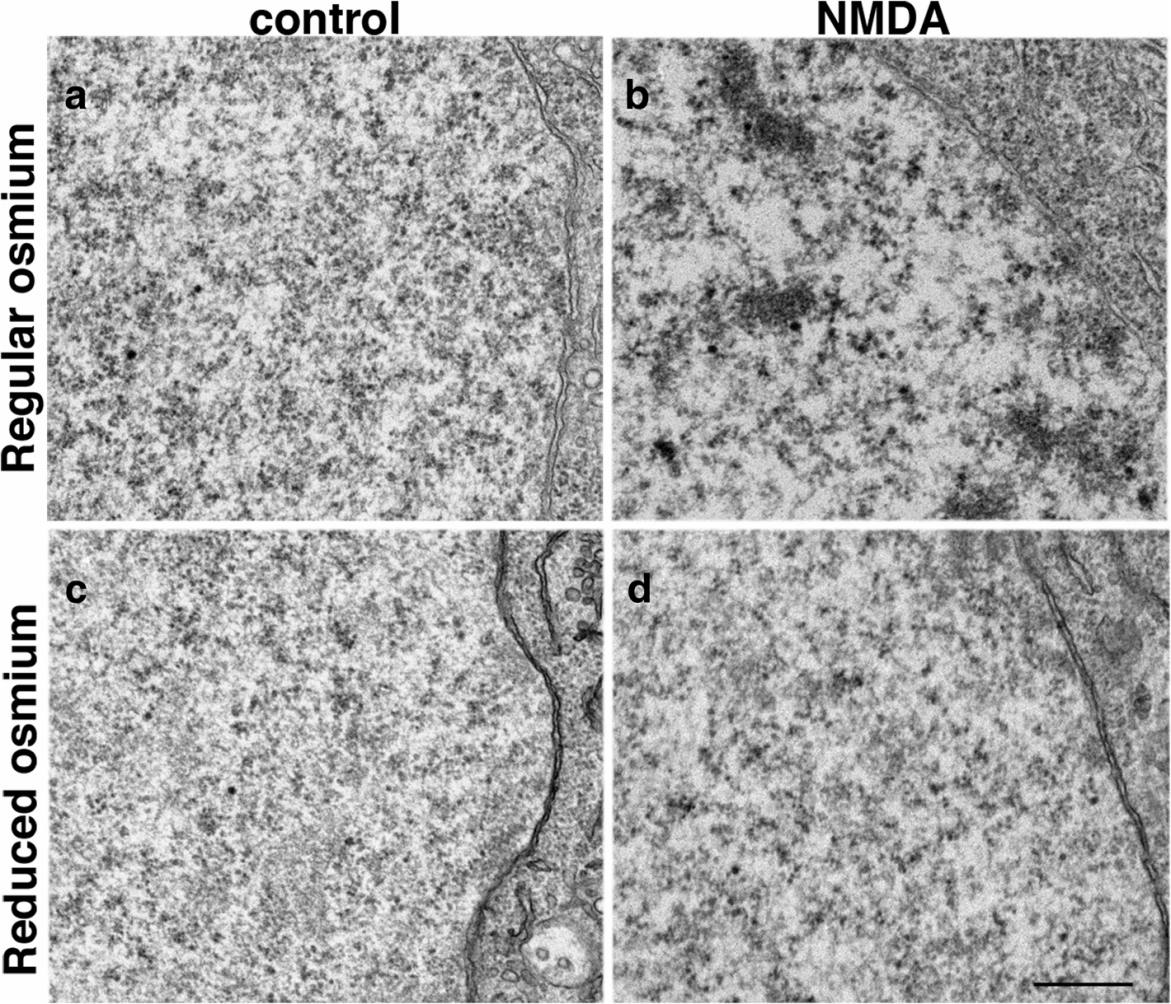
While neuronal chromatin appear non clustered under control conditions (**a**, **c**), NMDA-induced clustering of dark chromatin was prominent in neuronal nucleus from dissociated hippocampal cultures post-fixed with regular osmium tetroxide (**b**) but far less obvious in samples post-fixed with “reduced osmium” (1% ferrocyanide mixed with 1% osmium tetroxide, (**d**). Samples were sister cultures with the same NMDA treatment and processed in parallel. Scale bar = 0.5 μm

Likewise, in dissociated hippocampal neuronal cultures, neuronal nuclei had a non-clustered appearance under control conditions (Fig. 5a) in all 18 samples examined, but the chromatin consistently became clustered upon treatment with high K^+^ (11 samples; Additional file 1) or with NMDA (8 samples; Fig. 5b). Thus, in all three experimental systems studied here, this clustering of dark chromatin in neuronal nucleus is a reliable indicator that neurons are under heightened excitatory conditions.

Interestingly, this stimulation-induced clustering of the chromatin was readily detectible only when samples were stained with regular osmium tetroxide treatment (Fig. 5b), but much less noticeable with “reduced osmium” treatment (Fig. 5d; [12]) where osmium tetroxide was mixed with potassium ferrocyanide, indicating that the clustered chromatin or its associated histones was selectively stained upon regular osmium treatment but not upon “reduced osmium” treatment. Thus, absence of chromatin clustering in samples stained with “reduced osmium” is not an indication that neurons are at a basal state.

**Fig. 6:**
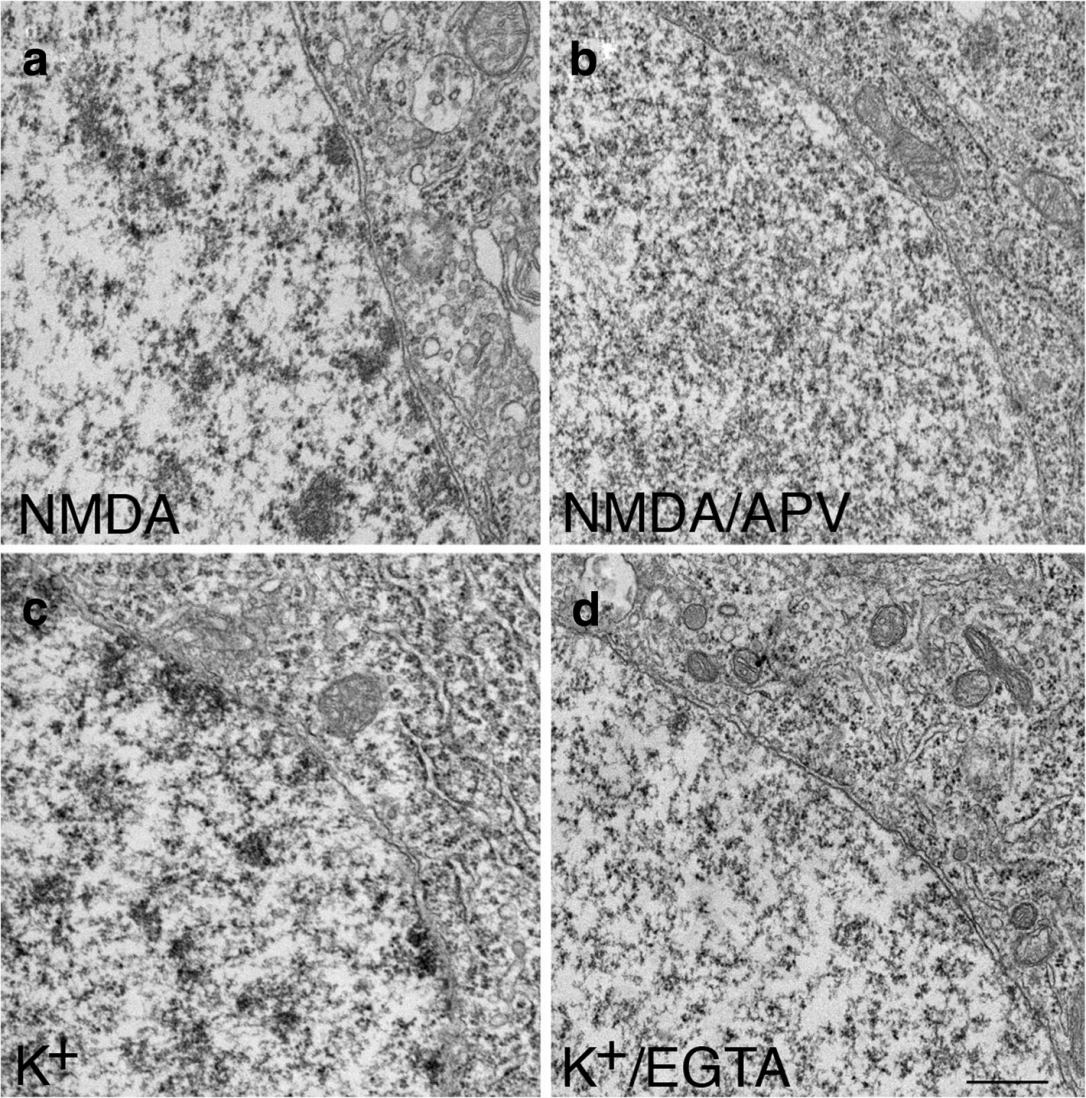
Stimulation-induced clustering of chromatin is calcium-dependent. In dissociated hippocampal cultures, the NMDA-induced clustering of chromatin (**a**) was blocked by APV (**b**). The depolarization-induced clustering of chromatin (**c**) was blocked by chelation of extracellular calcium with EGTA (**d**). Scale bar = 0.5 μm

The nuclear chromatin clustering induced by NMDA (Fig. 6a) in these dissociated hippocampal neurons was blocked by APV (Fig. 6b), an NMDA antagonist (3 exp). Since activation of NMDA receptors triggers calcium influx, the calcium-dependency of chromatin clustering was further tested. When the extracellular calcium was chelated by EGTA, the high K^+^-induced nuclear chromatin clustering (Fig. 6c) was indeed blocked (Fig. 6d; 3 exp).

### Stimulation-dependent formation of endoplasmic reticulum (ER) cisternal stacks in neuronal cytoplasm

**Fig. 7:**
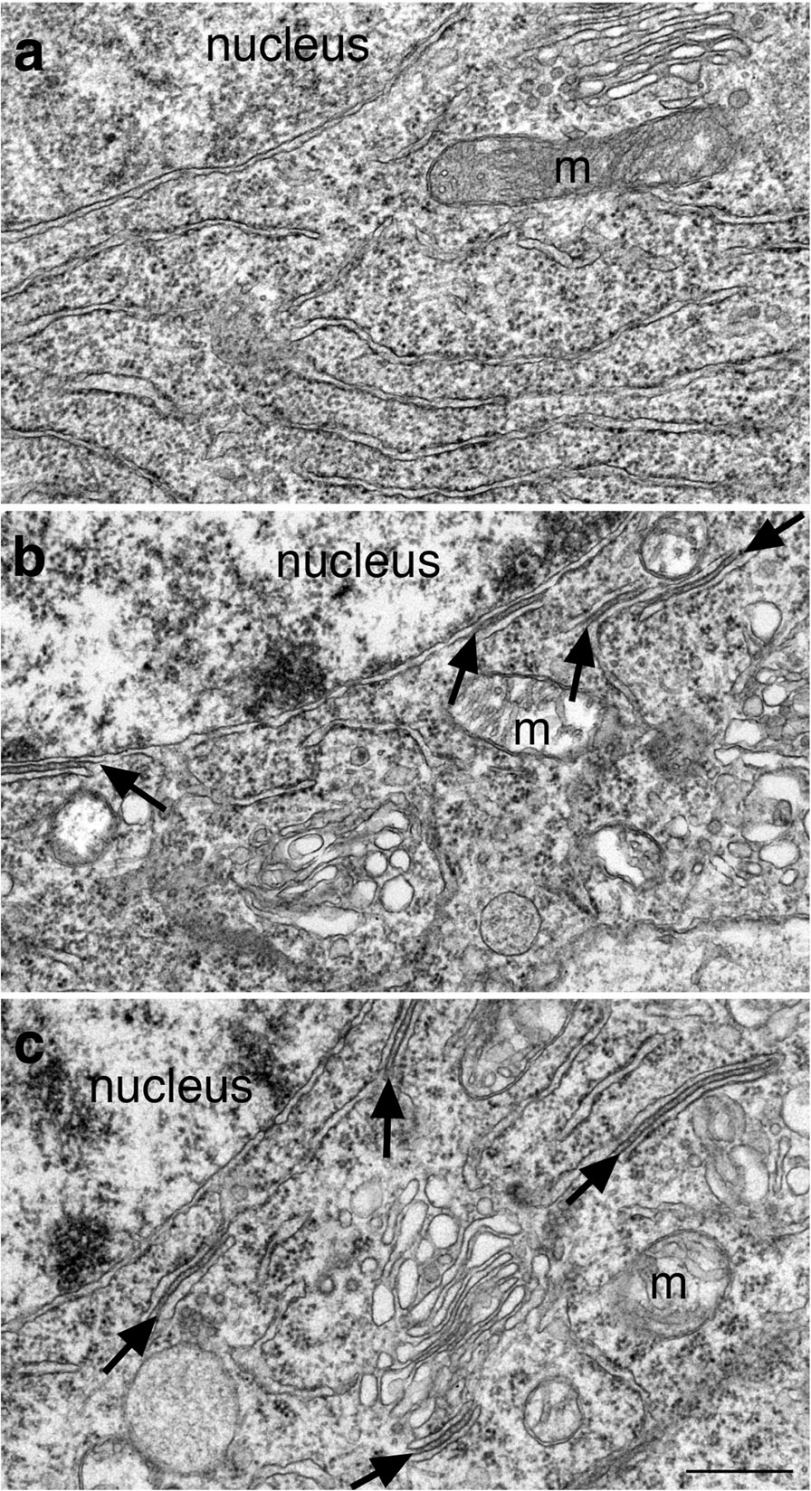
ER cisternal stacks (arrows in **b**, **c**) were absent in neuronal somas under control conditions (**a**), but appeared upon stimulation, such as depolarization with high K^+^ (90 mM, 3 min, **b**) or treatment with NMDA (50 μM, 2 min, **c**). Samples were from the CA1 region of the hippocampal slice cultures. Consistent with Fig. 3, the nuclei of these pyramidal neurons appeared non-clustered under control conditions (**a**), but the chromatin aggregated into dark clusters upon stimulation (**b**, **c**). Additionally, mitochondria (m) appeared swollen upon stimulation (**b**, **c**). Scale bar = 0.5 μm

ER cisternal stacks form in cerebellar Purkinje neurons under hypoxia-induced excitatory stress [4, 8]. Here, hippocampal slice cultures were used to determine if similar ER stacks form in forebrain neurons, and to examine the time course and reversibility of the formation of these ER stacks. As seen in Fig. 7a, ER stacks were absent in neurons under control conditions in all 15 samples, but were consistently prevalent in slice cultures treated for at least 2 min with high K^+^ (Fig. 7b) in 8 samples, or NMDA (Fig. 7c) in 10 samples. Thus, ER stacks indeed form in hippocampal neurons during heightened stimulation.

The stimulation-induced formation of ER cisternal stacks in hippocampal slice cultures progressed with treatment time as indicated by exp. 1 in Table 1 and exp. 1 and 2 in Table 3. There were only a few ER stacks at 30 s (0–14 per 100 neurons) to 1 min (6–57 per 100 neurons) of treatment, and ER stacks were more prevalent at 2–3 min (167–688 per 100 neurons). The formation of ER stacks was reversible as indicated by experiments 4 and 6 in Table 1, where ER stacks were no longer observed after the high K^+^ medium was washed out and samples were allowed to recover in control medium for the indicated periods of time. As expected, a longer treatment time (3 min in exp. 4 vs. 1 min in exp. 6) required a longer recovery time (10 min in exp. 4 vs. 5 min in exp. 6). Interestingly, as indicated in exp. 3 and 5, ER stack formation did not stop with the cessation of treatment, and ER stacks still continued to form 1–2 min after the washout of high K^+^ medium.

**Table 1:** Effect of depolarization with high K^+^ on number of ER cisternal stacks in neuronal somas in slice cultures, expressed as number per 100 neuronal somas

Exp	Cont	30″ K^+^	1′ K^+^	2′ K^+^	3′ K^+^	5′ K^+^	K^+^ + 1′ rec	K^+^ + 2′ rec	K^+^ + 5′ rec	K^+^ + 10′ rec	K^+^ + 1 h rec
1	0 (23)	14 (21)		241 (22)		659 (17)					
2	0 (21)				376 (21)						
3	0 (14)				167 (21)		305 (21)		20 (15)		
4	0 (22)				327 (22)					0 (27)	0 (24)
5	0 (19)		10 (21)					85 (13)			
6	0 (21)		57 (23)				55 (20)		0 (17)		
Mean ± SEM	0	27 ± 15		278 ± 46					7 ± 7		

Statistical analysis by ANOVA with Tukey’s post-test: data points from 30″ and 1′ K^+^, 2–3′ K^+^, and 5–10′ recovery were pooled, respectively (mean values of each listed in the last row). 2–3′K^+^ is highly significant over other conditions (*P* < 0.0001 vs. cont, 30′-1′K^+^, 5–10′ rec). Not significant among all other multiple comparisons including cont, 30′-1′K^+^ and 5–10′ rec

*(n)* number of somas examined

*rec* recovery in control medium

**Fig. 8:**
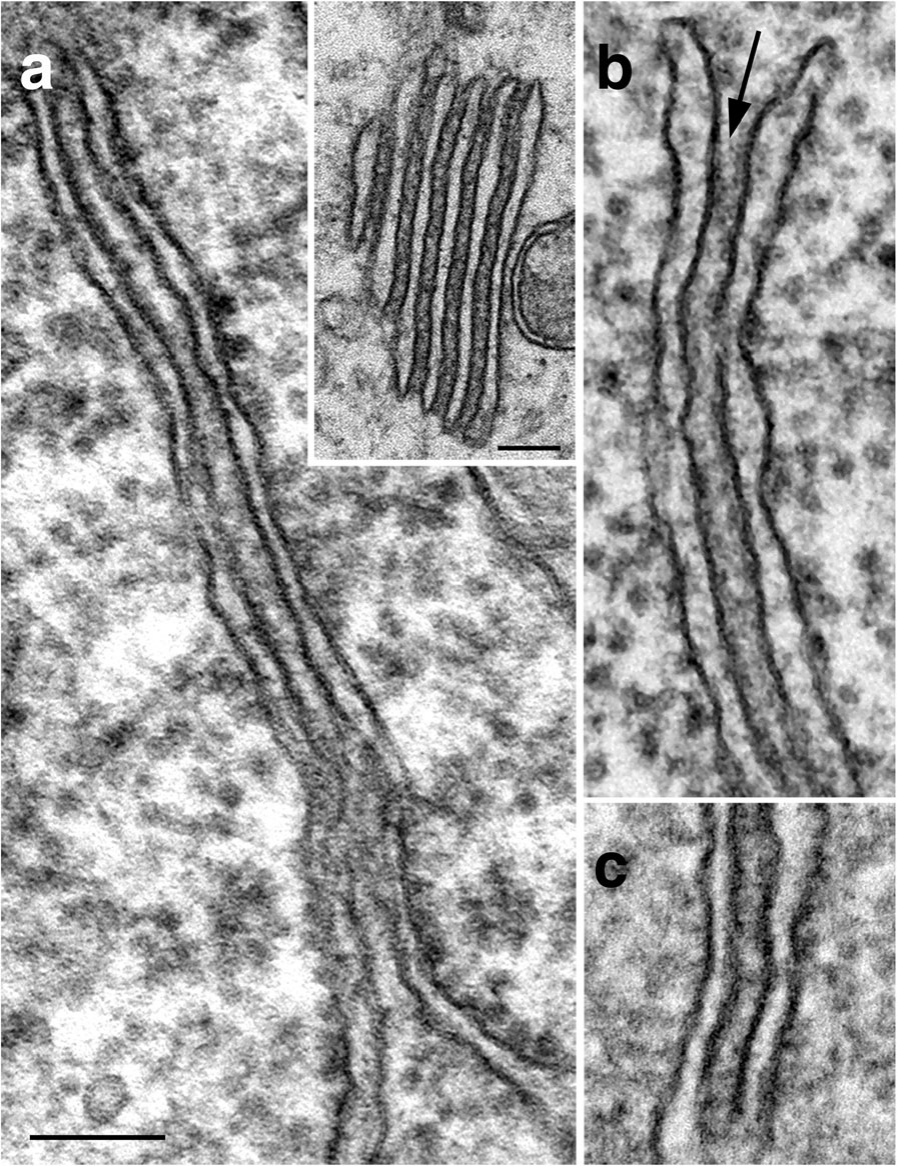
ER cisternal stacks from different experimental systems display the same configuration and gap width of ~ 13 nm (arrow in **b**). **a** Images from delayed perfusion-fixed brains: pyramidal neuronal soma of the CA1 region of the mouse hippocampus, and Purkinje dendrites of the rat cerebellum (inset in a). **b** Hippocampal slice cultures depolarized with high K^+^. **c** Dissociated hippocampal neurons treated with NMDA. Scale bar = 0.1 μm

The formation of neuronal ER cisternal stacks was stimulation-dependent in all three experimental systems examined here, with identical configuration and gap width (~ 13 nm), in brain (Fig. 8a), in organotypic slice cultures (Fig. 8b), and in dissociated cultures (Fig. 8c). The gap is typically filled with dense materials that sometimes appear with a periodicity [13].

In fast perfusion-fixed brains, no ER cisternal stacks were detected in any brain areas examined including cerebral cortex, hippocampus and cerebellum from one adult rat and twelve mice at ages ranging from 17 days to 3 months. In seven delayed perfusion-fixed rat and mouse brains, ER stacks were especially abundant in cerebellar Purkinje cells from soma to spines, and multiple stacks were common (Fig. 8a inset; [4]). These ER stacks of Purkinje neurons contain high concentrations of inositol 1, 4, 5-triphosphate receptors (IP_3_R) [13–15], a protein involved in calcium release from the ER lumen. Here in the present study, structurally identical ER stacks were present in hippocampus and cerebral cortex, but at much lower frequencies than those in Purkinje cells. Additionally, unlike in Purkinje somas, multiple stacks were rarely seen in the forebrain neurons. These observations are consistent with the overwhelmingly high levels of IP_3_R in Purkinje cells over other neuronal cell types [15, 16].

In dissociated hippocampal neuronal cultures, as in slice cultures, ER cisternal stacks were absent under control conditions, and only present when cells were treated with high K^+^ media (Table 2) or with NMDA (exp 3–5 in Table 3). However, upon identical treatment of 2 min of high K^+^ or NMDA, neurons in dissociated cultures typically formed fewer ER stacks (12 and 3% for high K^+^ or NMDA, respectively) than those in slice cultures. This stimulation-induced formation of ER stacks is calcium-dependent. No ER stacks were present when EGTA was included in the high K^+^ medium (exp 5–7 in Table 2).

**Table 2:** Effect of depolarization with high K^+^ on number of ER cisternal stacks in neuronal somas in dissociated cultures, expressed as number per 100 neuronal somas

Exp	Cont	2′ K^+^	3′ K^+^	2′K^+^/EGTA	3′K^+^/EGTA
1	0 (20)	20 (20)			
2	0 (31)	0 (16)			
3	0 (22)	83 (18)			
4	0 (20)	32 (28)	110 (20)		
5	0 (20)	15 (20)		0 (20)	
6	0 (20)	31 (16)	65 (17)		0 (20)
7	0 (20)		74 (23)		0 (20)
Mean ± SEM	0	30 ± 12	83 ± 14		0

Statistical analysis by ANOVA with Tukey’s post-test: 3′ K^+^ is significant over other conditions (*P* < 0.0005 vs. cont; *P* < 0.05 vs. 2′K^+^; *P* < 0.005 vs. 3′ K/EGTA). 2′ K^+^ is barely significant over control (*P* < 0.1). Other multiple comparisons are all non-significant

*(n)* number of somas examined

**Table 3:** Effect of NMDA treatment on number of ER cisternal stacks in neuronal somas in slice cultures (exp 1–2) as well as in dissociated cultures (exp 3–5), expressed as number per 100 neuronal somas

Slice exp	Cont	30″ NMDA	1′ NMDA	2′ NMDA	3′ NMDA
1	0 (17)	0 (20)	6 (16)	461 (18)	
2	0 (20)	5 (20)	55 (20)	289 (18)	688 (17)
Mean ± SEM	0	2.5 ± 2.5	31 ± 25	375 ± 86	
Cells exp	Cont			2′ NMDA	
3	0 (20)			25 (20)	
4	0 (20)			0 (20)	
5	0 (16)			10 (20)	
Mean ± SEM	0			12 ± 7	

Statistical analysis by ANOVA with Tukey’s post-test:

For slice culture experiments, 3′ NMDA is highly significant over other conditions (*P* < 0.0001 vs. cont, 30″NMDA, 1′NMDA; *P* < 0.005 vs. 2′ NMDA). 2′ NMDA is highly significant over other conditions (*P* < 0.0001 vs. cont; *P* < 0.0005 vs. 30′ NMDA, 1′ NMDA). Not significant among all other multiple comparisons including cont, 30″ NMDA and 1′ NMDA

For dissociated cell culture experiments, not significant between control and 2′NMDA

*(n)* number of somas examined

**Fig. 9:**
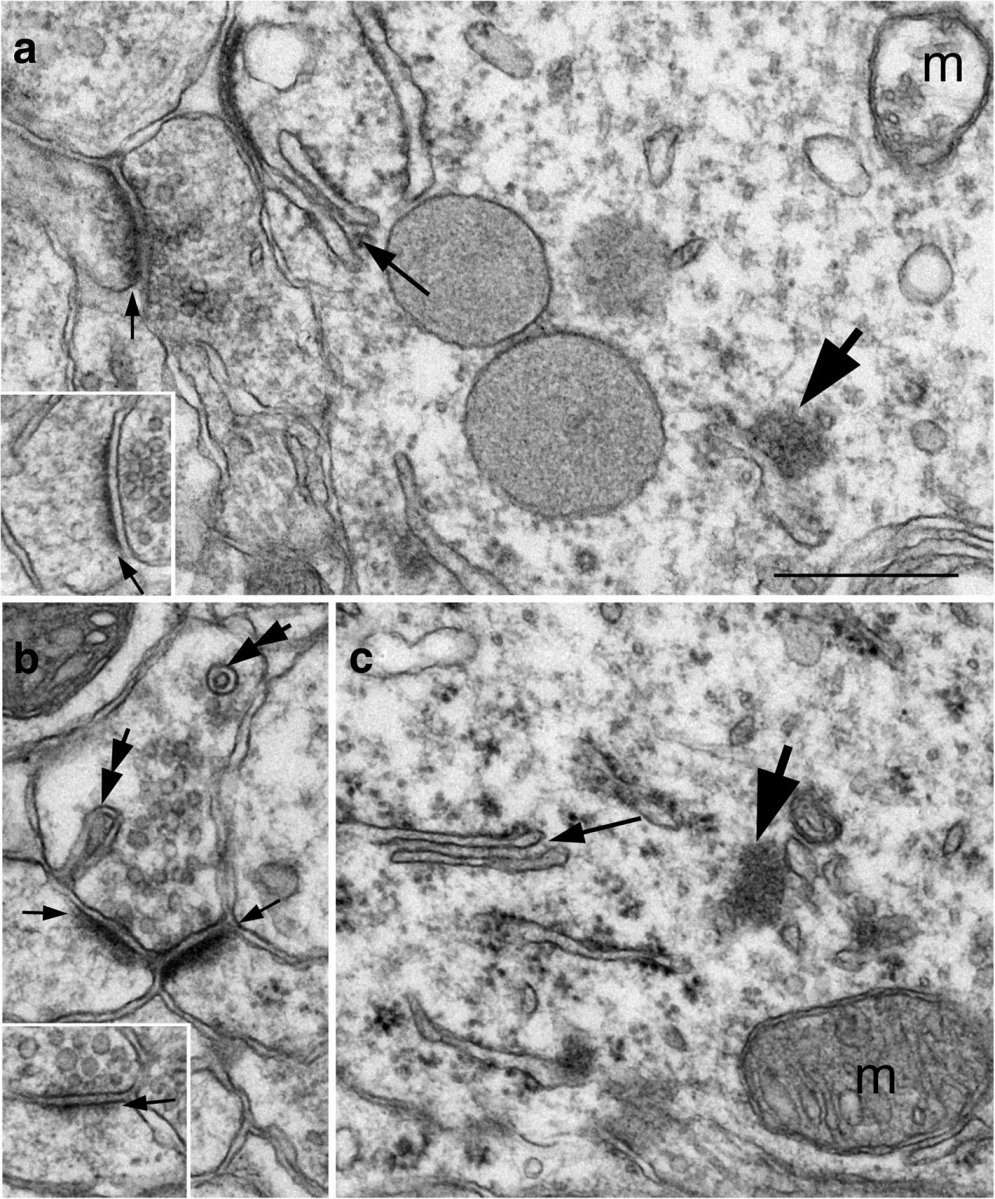
Images of the CA1 region of the hippocampus from a slice culture treated with 50 μM NMDA for 5 min (**a**), and from a delayed perfusion-fixed mouse brain (**b**, **c**). In addition to ER cisternal stacks (long small arrows), presence of CaMKII clusters (large arrows) and spinules (double arrows in **b**) indicates that these neurons are under heightened excitatory conditions. Furthermore, the thickness of PSD (short small arrows in **a**, **b**) is conspicuously greater than those of the control samples (inset of a is from a sister slice culture under control conditions; inset of b is from an age-matched mouse with fast perfusion-fixation). Mitochondria (m) appeared swollen in (**a**) but not in (**b**). Scale bar = 0.5 μm

Notably, whenever ER cisternal stacks were observed in neuronal somas, the nuclei of these neurons always contained clustered chromatin (cf. Fig. 7). Furthermore, ER cisternal stacks were consistently present along with other benchmarks for stimulation-induced structural changes (Fig. 9), including the formation of CaMKII clusters [4, 6, 17] and synaptic spinules [7], as well as an increase in thickness and curvature of PSD [3, 4]. These observations further verified that presence of ER cisternal stacks is a reliable indicator that neurons are under heightened excitatory conditions.

### Structural changes of neuronal mitochondria under different conditions

**Fig. 10:**
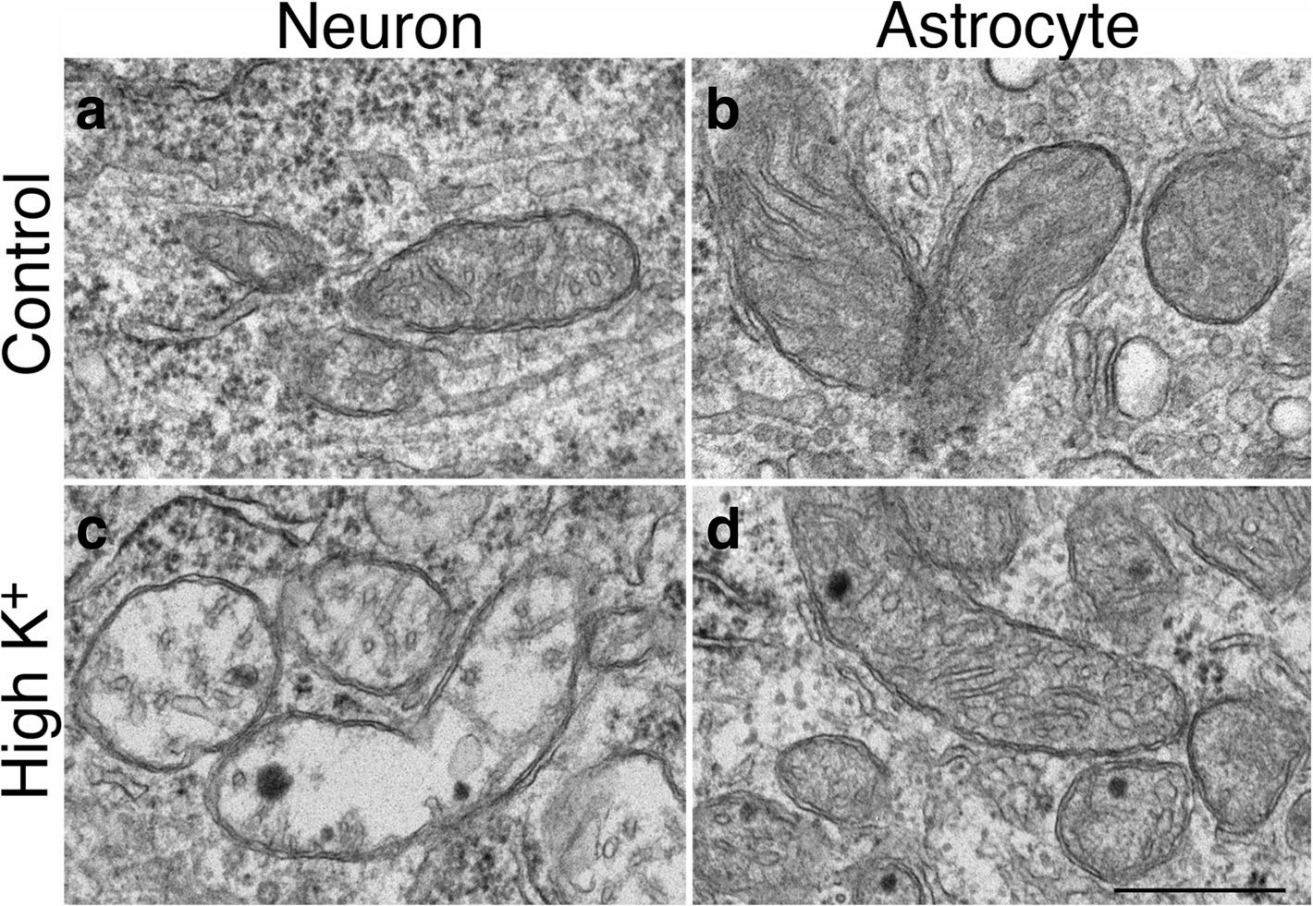
Mitochondria in neuronal somas became swollen upon depolarization (**c** vs. **a**; **c** was treated with 90 mM K^+^ for 3 min), while mitochondria in astrocytes displayed similar features under control conditions (**b**) or upon depolarization (**d**). Images of neurons and astrocytes were collected from the same samples of hippocampal slice cultures. Scale bar = 0.5 μm

**Fig. 11:**
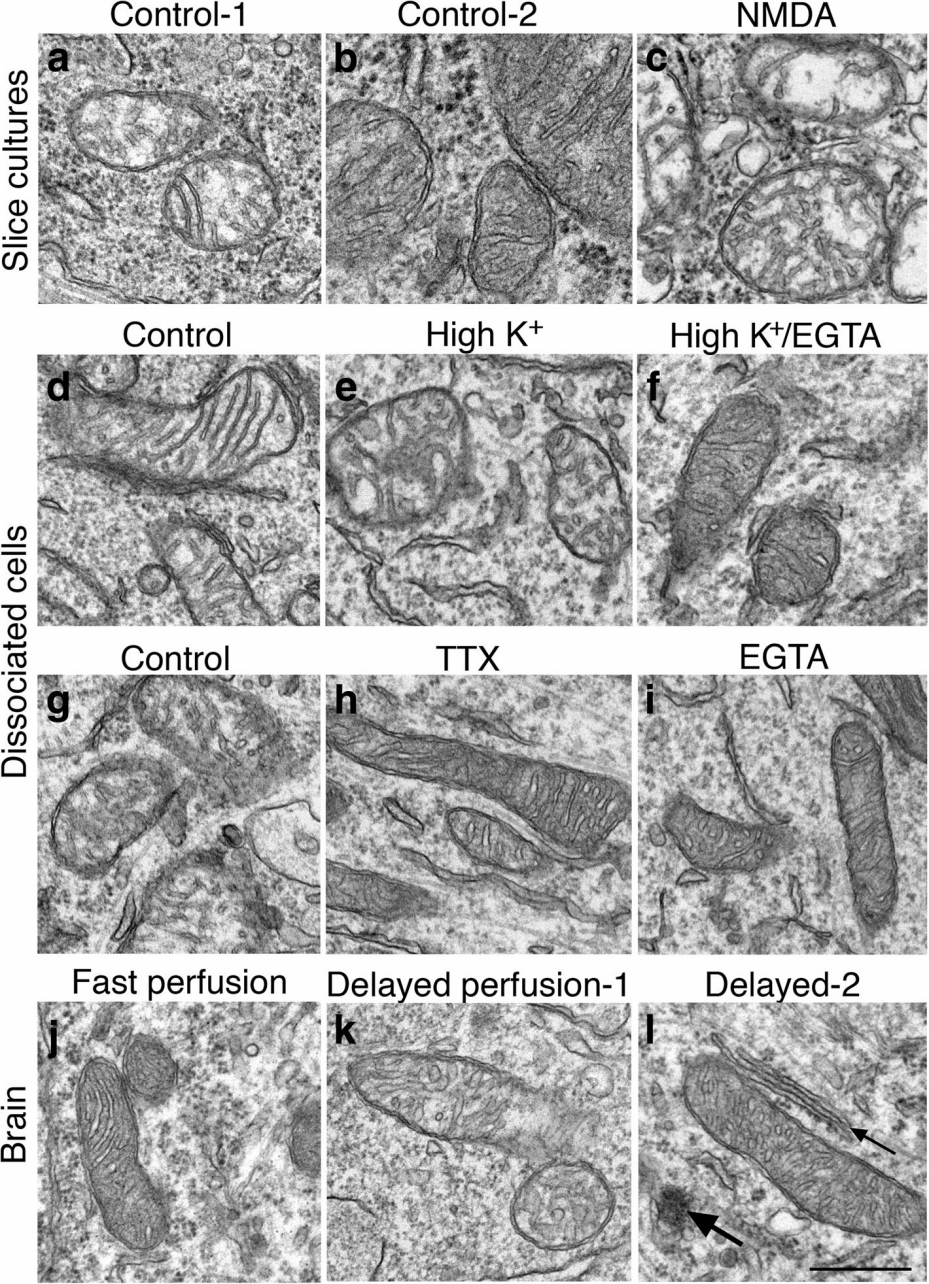
Structural change of mitochondria in three experimental systems under different conditions. In hippocampal slice cultures, mitochondria in control samples could appear swollen (**a**) or not (**b**) while those in NMDA-treated samples were consistently swollen (**c**). In dissociated hippocampal cell cultures, 55–90% of neurons contained swollen mitochondria in control samples (**d**). Upon 2–3 min depolarization with high K^+^, 87–100% neurons contain swollen mitochondria (**e**). When EGTA, a calcium chelator, is included in the high K^+^ medium, mitochondria were not swollen (**f**). When dissociated cell cultures were incubated for 1 h with or without TTX, 50–67% of neurons contained swollen mitochondria in control samples (**g**) while the great majority (more than 90%) of mitochondria in TTX-treated samples were not swollen (**h**). Washing control samples with EGTA for 2 min also prevented mitochondria from swelling (**i**). In perfusion-fixed mouse brains, the great majority of mitochondria in fast perfusion-fixed brains were not swollen (**j**), while some swollen mitochondria were seen in delayed perfusion-fixed brains (**k**). However, many mitochondria in delayed-fixed brains were not swollen (**l**), and co-existed with CaMKII clusters (large arrow in l) and ER cisternal stacks (small arrows in l), two structural benchmarks indicating that this neuron was under hypoxic excitatory stress. Scale bar = 0.5 μm

A third stimulation-induced structural change is that mitochondria in neuronal somas became swollen. In hippocampal slice cultures, virtually all mitochondria in neuronal somas appeared swollen upon high K^+^ (Figs. 7b, 10c and 11e) or NMDA (Figs. 7c and 11c) treatment. This structural change is specific to neurons as the ultrastructure of mitochondria in astrocytes did not change upon depolarization (Fig. 10b, d).

However, neuronal mitochondria in control samples often also appeared swollen in slice cultures (Fig. 11a) as well as in dissociated cell cultures (Fig. 11d, g). The swollen mitochondria in control samples were only seen in some neurons, and not all mitochondria in the same neuron were swollen to the same degree. For example, mitochondria at different degrees of swelling from control samples were shown in Fig. 11 a and b for slice cultures, and Fig. 11 d and g for dissociated neurons. To test the possibility that these control neurons containing the swollen mitochondria had higher basal level activity, TTX (plus APV and CNQX) was applied to dissociated cell cultures to suppress neuronal activity. Indeed, the great majority of mitochondria in TTX-treated samples were not swollen (Fig. 11h). In two experiments, the percent of cells displaying swollen mitochondria was 50 and 67% for control samples, and 0 and 8% for TTX-treated samples.

Interestingly, while examining the high K^+^-treated samples of dissociated cell cultures with or without EGTA, it became apparent that most mitochondria in neuronal somas in the presence of calcium were swollen (Fig. 11e) while those in the presence of EGTA were not (Fig. 11f). In 3 experiments, the average percentages of cells displaying swollen mitochondria in control, high K^+^ and high K^+^/EGTA samples were 67 ± 11, 92 ± 4 and 0%, respectively. The high K^+^/EGTA samples were significantly different from control and high K^+^ samples (*P* < 0.005 vs. control; *P* < 0.0005 vs. high K^+^; ANOVA with Tukey’s post-test). Thus, calcium influx during activity may be crucial in producing swollen mitochondria. In order to test whether the swelling in mitochondria in control samples can also be suppressed by blocking calcium influx, extracellular calcium was eliminated by treating the samples with calcium-free control media containing EGTA for 2 min. Indeed, mitochondria were not swollen (Fig. 11i). Altogether, these observations suggest that neuronal mitochondria appear to be an extremely sensitive indicator for the activity state of neurons in slice as well as in dissociated cultures, and that the swelling of mitochondria is dependent on calcium influx.

When perfusion-fixed brains from two sets of matched animals were scored for mitochondria morphology, relatively few mitochondria were swollen compared to those of in vitro samples. The great majority of mitochondria were not swollen in fast perfusion-fixed brains (Fig. 11j), and percentages of neurons containing swollen mitochondria were 0 and 7%. In two delayed perfusion-fixed brains, 22 and 33% of neurons contained swollen mitochondria (Fig. 11k) while the majority of mitochondria were not swollen (Fig. 11l). However, swollen mitochondria were consistently seen in poorly perfusion-fixed brains where fixative was not effectively delivered via blood vessels to surrounding tissues, evidenced by presence of blood cells in collapsed vessels.

## Discussion

The present study describes three stimulation-induced structural changes in neurons that are easily detectible: clustering of nuclear chromatin, formation of ER cisternal stacks in the cytoplasm, and swelling of mitochondrial matrix. The first two structural benchmarks are unequivocal and useful indicators that neurons are under heightened excitatory conditions, while the swollen mitochondria could be induced by basal levels of activity. Excitatory conditions can be produced inadvertently during experimental manipulations such as transfer and mechanical handling of cell cultures. This consideration is especially important when interpreting results from perfusion-fixed brains, where the procedure of perfusion itself, if not performed well, may introduce ischemic stress, and possibly induce excitotoxicity [4].

Physiological consequences of the described stimulation-induced structural changes are at present largely undefined. Structural changes in chromatin have been linked to development and activity [18, 19], including long-term potentiation [20]. Additionally, upon 20 min of transient global ischemia, neuronal nuclei displayed chromatin clustering, which is reversed 3 h after reperfusion [21]. Of particular interest is the resemblance of the nuclear structural change induced by ischemia to the acute stimulation-induced chromatin clustering presented here. Although the earliest time point was set at 20 min after ischemia [21], chromatin clustering may have started sooner. Indeed, it is shown here that a delay of a few minutes in perfusion fixation that mimics ischemia [4] induced chromatin clustering in neuronal nuclei. Furthermore, in hippocampal slice cultures, 30 s of stimulation already induced chromatin clustering. Activity-dependent movement of postsynaptic proteins into the nucleus may trigger changes in gene expression [22, 23]. However, signaling via transport of synaptic proteins seems too slow to explain the current results. A more likely candidate to mediate such a rapid, activity-dependent nuclear response is calcium signaling. One consequence of depolarization and NMDA receptor activation is a rapid rise in intracellular calcium concentration, which is easily equilibrated across the nuclear membrane, and can trigger calcium-dependent transcriptional regulation in the nucleus [22, 24]. Indeed, the present study demonstrated that the stimulation-induced chromatin clustering in the neuronal nucleus is calcium-dependent. It is generally assumed that dark chromatin is transcriptionally inactive while loosely arranged chromatin in the lighter space of the nucleus may be transcriptionally active [18]. Accordingly, the stimulation-induced clustering of dark chromatin presented here may cause certain genes to become less accessible for transcription.

A second stimulation-induced structural benchmark presented here is the ER cisternal stacks. In Purkinje cells, ER stacks contain high concentrations of IP_3_R on the side of the ER membranes that face the narrow gap, while the side of the ER membranes that face the cytoplasm have low levels of IP_3_R [13, 15]. Interestingly, the uniform gap of the ER stack is filled with dark material, suggesting that protein interactions may tether the two membranes, potentially restricting the diffusion of molecules between the gap and the cytoplasm. Since IP_3_R are involved in IP_3_-induced calcium release from the ER to the cytoplasm, formation of ER stacks that localizes IP_3_R in this pattern may restrict the receptor’s accessibility to cytosolic IP_3_, thereby reducing calcium release into the cytoplasm. Importantly, the stimulation-induced formation of ER stacks is calcium-dependent and progresses with treatment time, correlating with a rise in intracellular calcium concentration. Thus, formation of ER stacks could prevent further calcium overload during heightened stimulation by restricting calcium release from ER to the cytoplasm.

These stimulation-induced ER stacks resemble another ER structural specialization, termed lamellar bodies [25, 26]. Both structures are composed of stacks of ER with a uniform gap between stacks, and lack ribosomes in the membranes facing the gaps. However, the two structures display different spacing: the gap width is ~ 13 nm for the ER stacks, and 30 nm for the lamellar bodies in adult animals [25, 26] (Additional file 3). This difference in gap width suggests that different proteins may be present in the gaps tethering the membranes of these two structural specializations of the ER. Additionally, the ER cisterns in lamellar bodies can become flattened while those in ER stacks never do. Furthermore, lamellar bodies are present in control samples (Additional file 3) while ER stacks are not.

The time course of formation and recovery of the two stimulation-induced structural change reported here are different in slice cultures. Chromatin clustering was consistently apparent at 30 s of stimulation while the formation of ER stacks typically needed a minute or more to become readily detectible. On the other hand, the recovery of ER stacks was faster than the chromatin clustering. For example, upon 5 min of recovery following 1 min of high K^+^ treatment, ER stacks were no longer present while nuclear chromatin clustering still partially remained. Thus, these two calcium-dependent structural changes respond differently to the rise and fall of calcium, and the formation of ER stacks probably requires a higher threshold of calcium concentration.

A third finding here is that neuronal mitochondria from in vitro samples became swollen upon stimulation, consistent with a report on calcium-dependent mitochondria changes upon excitotoxic NMDA treatment [9]. However, neurons in control samples also contained a noticeable number of swollen mitochondria which could be blocked by suppressing basal activity or by eliminating calcium influx. Thus, neuronal mitochondria in tissue culture systems are very sensitive to basal activity and calcium entry. Very few swollen mitochondria were seen in fast perfusion-fixed brains. It is possible that the neurons in deeply anesthetized animals had lower activity than control neurons in tissue culture. Surprisingly, even in delayed perfusion-fixed brains where neurons were under hypoxic excitatory stress, only ~ 30% of neurons contained swollen mitochondria, in contrast to the > 90% of neurons with swollen mitochondria in the stimulated in vitro samples. Considering that the delay in perfusion was 5–8 min, a time that is longer than the 2–3 min treatment time for the in vitro samples, it is possible that the calcium regulation and its effects on mitochondria are different at these different time points. Alternatively, an ischemia-like stress in brain could produce different effects on mitochondria than the heightened stimulations applied to neurons in culture. It is also possible that mitochondria in neurons maintained in vitro are more vulnerable to activity-induced swelling. Notably, in poorly perfusion-fixed brains where fixative was not effectively delivered to surrounding tissues, all neuronal mitochondria were swollen. Neurons in these samples likely underwent a longer period of hypoxic stress before they were fixed. This finding is consistent with the report that mitochondria were swollen after 30 min of ischemia [27].

The stimulation-induced structural changes presented here consistently co-existed with other structural benchmarks including CAMKII clusters, synaptic spinules, and increase in thickness and curvature of the PSD, that are induced by neuronal activity [3, 4, 7]. Interestingly, the formation of many of these stimulation-induced structural changes is calcium-dependent [6, 28] and may also offer protection against calcium overload during heightened activity. For example, stimulation-induced formation of CaMKII clusters with tightly bound molecules may limit the access of this enzyme to substrate [17], and a decrease in ER and plasma membrane contact area may limit calcium influx during intense activity [5]. The present study added two more reliable structural benchmarks, chromatin clustering and ER cisternal stacks, that are induced by stimulation in forebrain neurons. The present study also demonstrated that the morphology of mitochondria from neurons in tissue culture is affected by basal level of activity and calcium influx. These stimulation-induced structural changes may provide new insights into the neuron’s response to intracellular calcium rise.

## Additional files

Additional file 1: Neural chromatin clustering upon stimulation. (PDF 2492 kb)

Additional file 2: Chromatin clustered upon depolarization in neurons but not in astrocytes. (PDF 5705 kb)

Additional file 3: Structural differences between ER lamellar bodies and ER cisternal stacks. (PDF 3994 kb)
