# Supplementary material for: Stimulation-induced structural changes at the nucleus, endoplasmic reticulum and mitochondria of hippocampal neurons
Source: Mol Brain. 2018 Jul 27;11:44. doi: 10.1186/s13041-018-0387-2 (PMC6062868; doi:10.1186/s13041-018-0387-2)
Supplement: Supplementary file 1 — Neural chromatin clustering upon stimulation. (PDF 2492 kb) [file 13041_2018_387_MOESM1_ESM.pdf]

Additional file 1. Neuronal chromatin clustering upon stimulation

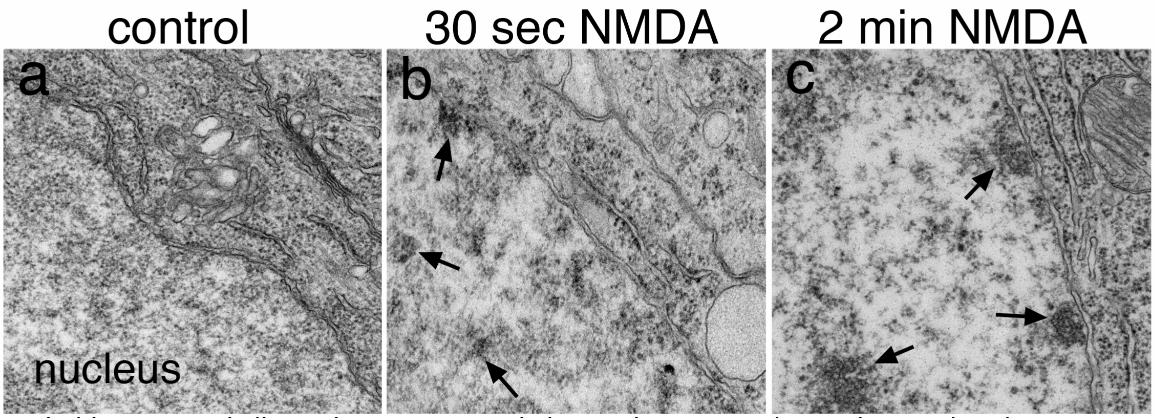

In hippocampal slice cultures, neuronal chromatin appeared non-clustered under control conditions (a), but became slightly aggregated into dark clusters (arrows) after 30 seconds of NMDA treatment (b), and this clustering progressed with time (c).

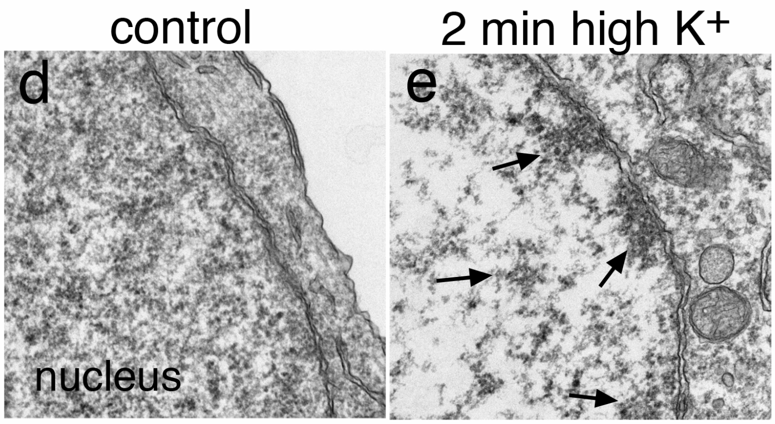

Similarly, in dissociated hippocampal cultures, neuronal chromatin appeared non-clustered under control conditions (d), but became clustered upon depolarization (e).
