# Supplementary material for: Stimulation-induced structural changes at the nucleus, endoplasmic reticulum and mitochondria of hippocampal neurons
Source: Mol Brain. 2018 Jul 27;11:44. doi: 10.1186/s13041-018-0387-2 (PMC6062868; doi:10.1186/s13041-018-0387-2)
Supplement: Supplementary file 2 — Chromatin clustered upon depolarization in neurons but not in astrocytes. (PDF 5705 kb) [file 13041_2018_387_MOESM2_ESM.pdf]

Additiona file 2. Chromatin clustered upon depolarization in neurons but not in astrocytes.

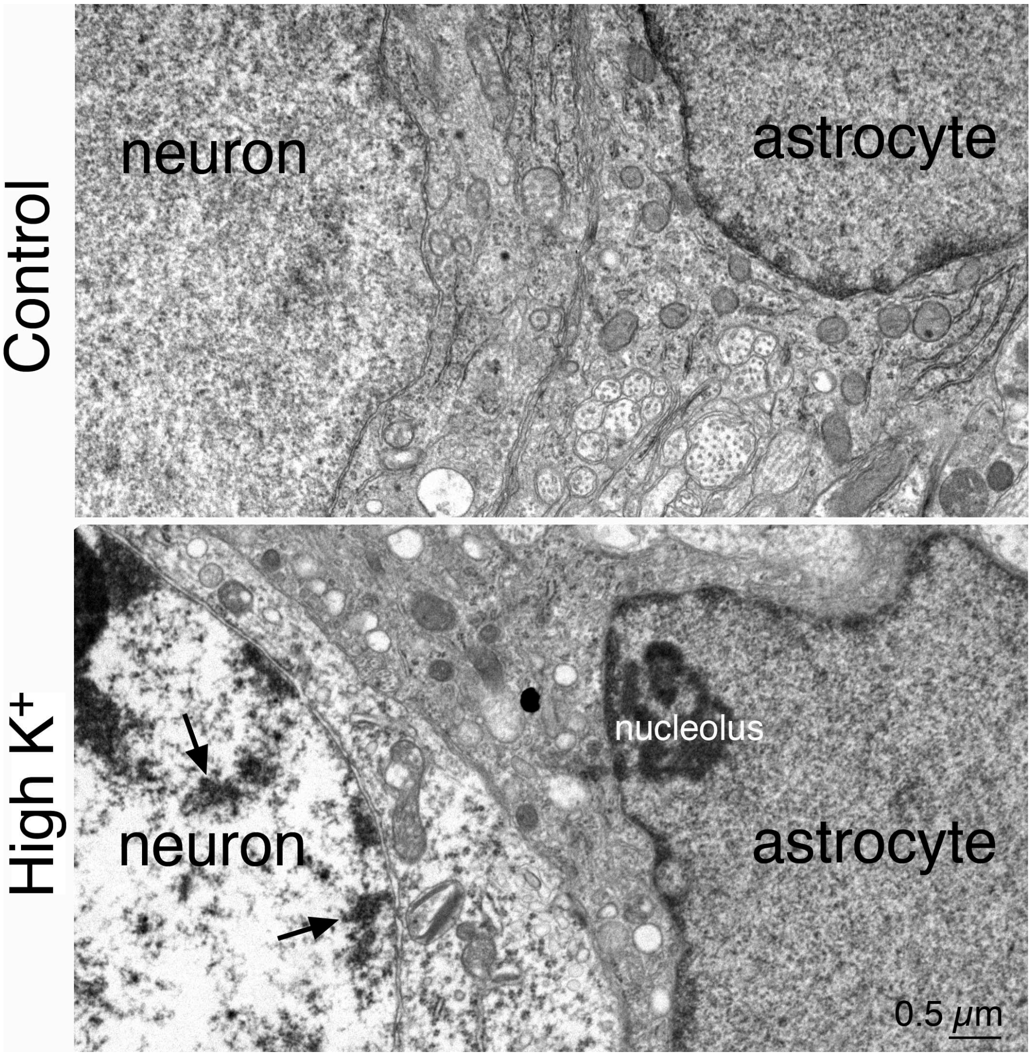

In hippocampal slice cultures under control conditions, neuronal chromatin appeared non-clustered while the chromatin in astrocytes showed a dark rim of irregular thickness. Upon treatment with high K^+ (90 mM for 3 min), neuronal chromatin formed dark aggregates (arrows) while no structural changes were detected in the nucleus of astrocytes. Scale bar = 0.5 μm.
