# Supplementary material for: Stimulation-induced structural changes at the nucleus, endoplasmic reticulum and mitochondria of hippocampal neurons
Source: Mol Brain. 2018 Jul 27;11:44. doi: 10.1186/s13041-018-0387-2 (PMC6062868; doi:10.1186/s13041-018-0387-2)
Supplement: Supplementary file 3 — Structural differences between ER lamellar bodies and ER cisternal stacks. (PDF 3994 kb) [file 13041_2018_387_MOESM3_ESM.pdf]

Additional file 3. Structural differences between ER lamellar bodies and ER cisternal stacks.

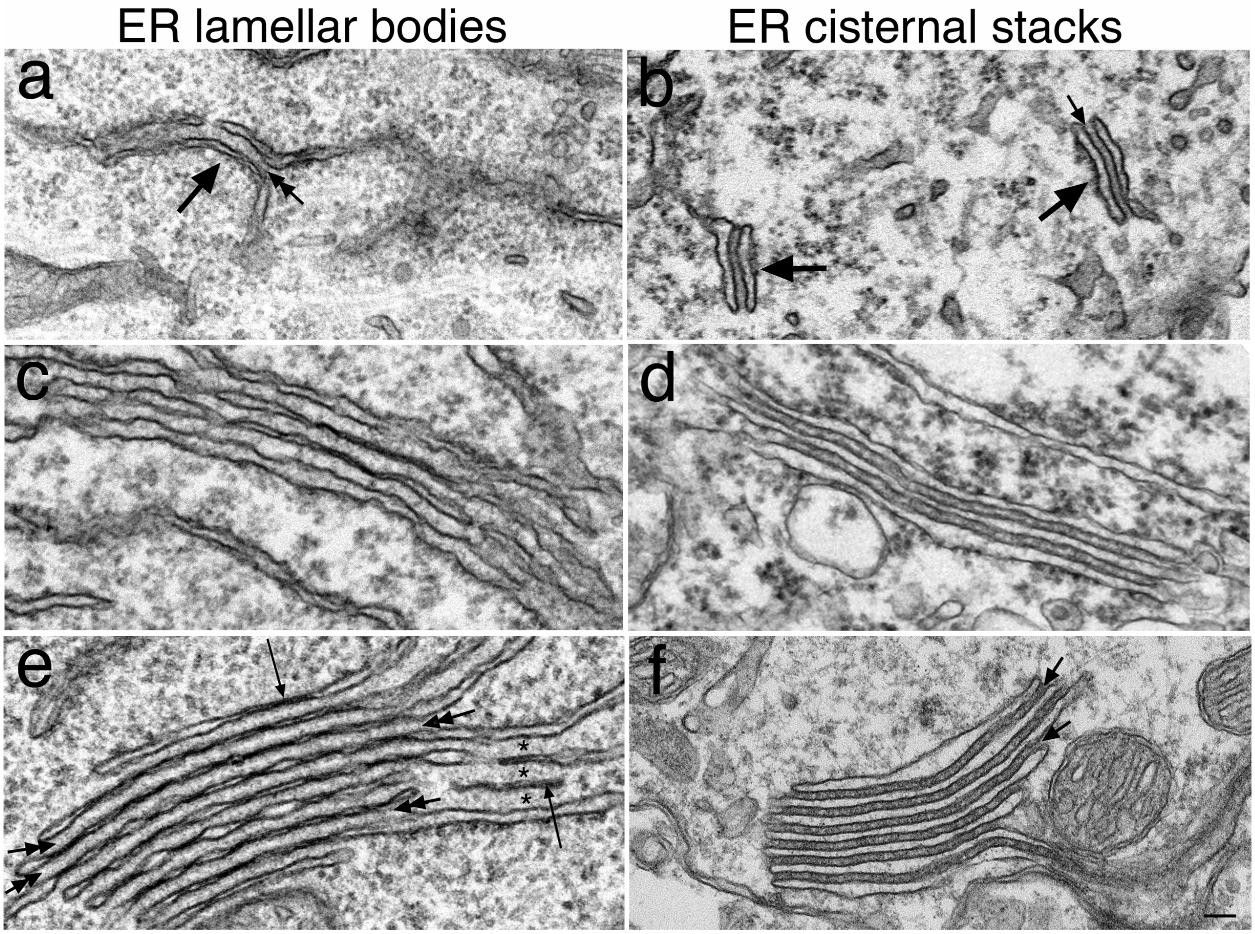

Neuronal ER can form "lamellar bodies" that contain from two layers of ER (a) to multiple layers (c, e) of ER cisterns. Images in a, c, e are from dissociated hippocampal neurons under control conditions (a), after 3 min of high K^+/EGTA in calcium-free conditions (c), and under recovery conditions (e, sample was treated with high K^+ for 2 min and then recovered for 30 min in control media). The gap width of the lamellar bodies is typically at 30 nm (double arrows in a, e) but can be wider at places (* in e) in neurons of dissociated or slice cultures. The lumen of ER lamellae can be open or flattened (long arrows in e). These features of lamellar bodies are similar to those of subsurface cisterns at the ER-plama membrane contact area [5].

In contrast, ER cisternal stacks are only found in neurons under heightened excitatory conditions, but never in neurons under resting conditions. (b) Two small ER stacks (large arrows) in a neuron of dissociated culture after 2 min of high K^+ treatment. (d) A multi-layered stack of ER in a hippocampal slice culture treated with 3 min of NMDA at 50 μM. (f) A multi-layered stack of ER in a cerebellar Purkinje dendrite from a delayed perfusion-fixed rat brain. The gap width is constant at ~13 nm (small arrows in b, f), and the lumen of these ER stacks is never flattened. Scale bar = 0.1 μm.
